# Editorial: Next Generation User-Adaptive Wearable Robots

**DOI:** 10.3389/frobt.2022.920655

**Published:** 2022-06-22

**Authors:** Thomas C. Bulea, Nitin Sharma, Siddhartha Sikdar, Hao Su

**Affiliations:** ^1^ Neurorehabilitation and Biomechanics Research Section, Rehabilitation Medicine Department, National Institutes of Health Clinical Center, Bethesda, MD, United States; ^2^ Joint Department of Biomedical Engineering, North Carolina State University and University of North Carolina-Chapel Hill, Raleigh, NC, United States; ^3^ Department of Bioengineering, George Mason University, Fairfax, VA, United States; ^4^ Center for Adaptive Systems of Brain-Body Interactions, George Mason University, Fairfax, VA, United States; ^5^ Department of Mechanical and Aerospace Engineering, North Carolina State University, Raleigh, NC, United States

**Keywords:** exoskeleton, wearable robot, rehabilitation, human-in-the loop, functional electric stimulation

Wearable robots, including powered exoskeletons and robotic prostheses, have created new possibilities for mobility augmentation and restoration among individuals with a variety of movement disorders, including spinal cord injury, stroke, amputation, and other neurological conditions (Esquenazi et al., 2017; Rodríguez-Fernández et al., 2021). Exoskeleton technology has progressed to create utility for unimpaired individuals by supporting load carriage, reducing joint loading or improving metabolic efficiency (Sawicki et al., 2020). Despite this progress, exoskeleton deployment in real-world, community environments remains limited. While there are multiple barriers to ubiquitous exoskeleton use, a key lynchpin is development of robust control systems that adapt to user intent, support the variety of mobility tasks that may be encountered, and account for variation in the user’s voluntary effort across such tasks.

This Research Topic highlights recent advances toward the development of such adaptive control systems for wearable robotics. The Research Topic presents three manuscripts detailing design and evaluation of hybrid exoskeletons combining functional electrical stimulation (FES) with powered exoskeletons, one that was evaluated in individuals with spinal cord injury (Nandor et al.) and two that were validated in able-bodied individuals (Molazadeh et al.; Chang et al.). One study presents a pediatric exoskeleton that provides adaptive assistance to knee extension to alleviate crouch and its evaluation in a child with cerebral palsy (Chen et al.). Two manuscripts present novel controllers which leverage reinforcement learning and their evaluation in simulation: one for assisting squatting motion (Luo et al.) and one for bipedal exoskeleton walking in three dimensions (Liu et al.). The final manuscript evaluates the fusion of surface electromyography (EMG) and muscle sonography to estimate limb movement in a variety of locomotor tasks (Rabe and Fey).

Hybrid exoskeletons are an attractive option for use in individuals with paralysis because they can potentially provide the therapeutic benefits of muscle activation and the enhanced mobility from electromechanical actuators. These systems fit particularly well with the focus of this research topic; FES-induced muscle fatigue results in unpredictable torque output from stimulated muscles and thereby necessitates an adaptive approach for supplementing motion with actuators. In this Research Topic, Nandor et al. introduce a novel Motor Assisted Hybrid Neuroprosthesis (MAHNP) with actuated hip and knee joints and a distributed control architecture that integrates the exoskeleton with customized FES systems. A supervisory gait event detector split the gait cycle into four discrete states. The hip and/or knee motors could be activated with bursts of torque to assist the stimulation-driven limb motion. The system was evaluated in two participants with SCI, each with different implanted stimulation systems. Each showed improvements in step length and gait speed when walking with exoskeleton assistance, demonstrating the utility of this muscle first hybrid approach. Molazadeh et al. introduced a robust adaptive strategy for shared control in a hybrid exoskeleton. The strategy used a neural network-based iterative learning controller combined with model predictive control to adaptively distribute torque generation between surface FES and motors at the knee. When evaluated in a sit-to-stand task, this shared control paradigm improved joint trajectory tracking at the hip and knee across multiple task repetitions, demonstrating the utility of the iterative learning process in allocating torque generation between FES and motors. The controller was stable and robust despite disturbance uncertainty, suggesting its future utility across a variety of movements. In yet another approach, Chang et al. implemented a combination of different controllers for cooperative use of FES and electric motors at the hip and knee for trajectory tracking during treadmill walking. The novelty here was combining trajectory tracking *via* FES and stiffness tracking using a cable-driven exoskeleton at the knee, while only motorized assistance was used at the hip. The controller was evaluated in three able-bodied individuals and showed capability to track target trajectories at both low and high speeds.

Chen et al. present a robotic exoskeleton designed for ambulatory children with crouch gait, another pathology that fits well with the aims of this topic. Rather than replacing lost function, the goal of this exoskeleton was assistance and rehabilitation. They introduced an adaptive controller that provided knee extensor torque proportional to the user’s instantaneous volitional effort. Their evaluation in one child with cerebral palsy showed the adaptive control improved crouch with less reduction in gait speed compared to non-adaptive assistance.

Luo et al. introduce design and numerical simulation of their reinforcement-based learning controller for cooperative assistance during a squatting motion. They use a multi-layer perceptron neural network, trained on a reference squatting motion with and without perturbations. The neural network outputs target joint positions, which are tracked by the actuators at the hip, knee and ankle using a proportional derivative controller. The controller was verified in multiple simulations that included perturbations and human-exoskeleton interactions, suggesting this approach may be extendable to other movements. However, they offer a word of caution that their modeling only included a passive muscle response. Thus, future work should focus on verification in users who retain some ability to generate movements themselves. Liu et al. paired reinforcement learning with two different artificial neural network controllers and evaluated them in a three-dimensional bipedal model of a human and exoskeleton. Their results showed a global neural network controller that encompasses the entire walking motion, versus local controllers for stance and swing, provided the most stable control. A key implication is their architecture doesn’t require a detailed dynamic model of the exoskeleton user, potentially expediting the transition of this controller into practice.

Rabe and Fey present a novel method of fusing surface EMG with sonography to continuously predict hip, knee, and ankle joint angles and velocities. Their results show that this fusion improves tracking during various walking tasks, including level ground, stair, and ramp ambulation. This approach holds promise for future robotic controllers as it enables movement tracking without instrumenting individual joints.

In summary, we believe this research topic contributes to the existing literature regarding adaptive control methods for wearable robotics and will pave the way for future advancements toward real-world functionality. Continued advancements in deep neural networks and machine learning (Laschowski et al., 2021), switched control theory (Sheng et al., 2021), and bioinspired control algorithms (Alibeji et al., 2018) have potential to address the challenges remaining for intent-detection, environment classification and exoskeleton control during various locomotion modes. Although the focus of this editorial was on mobility, the approaches introduced here may be applicable in upper extremity (UE) exoskeletons as well. Similarly, previously developed methods for robust and adaptive control of UE exoskeletons (Kiguchi et al., 2005; Brahmi et al., 2018) could be explored in lower limb exoskeletons. In addition, multi-modal approaches including those that leverage ultrasound imaging for intent prediction (Dhawan et al., 2019; Zhang et al., 2021) and muscle in the loop control of exoskeletons can increase adaptability during real-world use.
